# Supplementary material for: Fine Mapping of Candidate Gene Controlling Anthocyanin Biosynthesis for Purple Peel in Solanum melongena L
Source: Int J Mol Sci. 2024 May 11;25(10):5241. doi: 10.3390/ijms25105241 (PMC11121509; doi:10.3390/ijms25105241)
Supplement: Supplementary file 1 [file ijms-25-05241-s001.zip › ijms-2800930-supplementary.pdf]

Supplementary data

Table S1. Resequencing comparison results of pools

Samples	Clean reads (bp)	Clean reads (%)	Mapped reads rate (%)	Coverage (%)	Depth (×)
P_1	23041153050	99.85	99.94	98.20	19.71
P_2	22810874250	99.87	99.80	98.15	19.45
Green pool	45340847700	99.86	99.83	98.95	38.65
Purple pool	45636314250	99.84	99.87	98.97	38.85

Table S2. Variant types of genes in candidate regions

SNP/InDel Position	Ref	Alt	Al	Func	Gene ID
19201065	T	A	Exon	synonymous SNV	EGP22360
19201131	G	T	Exon	synonymous SNV	EGP22360
19234181	T	C	Exon	nonsynonymous SNV	EGP22361
19234457	C	T	Exon	nonsynonymous SNV	EGP22361
19257428	A	G	Exon	nonsynonymous SNV	EGP22363
19336092	G	GCT	Exon	insertion	EGP22369
19346652	C	T	Exon	nonsynonymous SNV	EGP22372

Note: 'Ref' represents SNP/InDel genotypes in reference genome; 'Alt' represents SNP/InDel genotypes in non-reference genome; 'Al' represents the functional position of SNP/InDel in candidate genes; 'Func' represents mutation type.

Table S3. Primers for KASP markers

Primer Name	Sequence	Chromosome
KA002375-X	GAAGGTGACCAAGTTCATGCTACTCAATCACTTTCATTTGCTTCG	Chr4
KA002375-Y	GAAGGTCGGAGTCAACGGATTACTCAATCACTTTCATTTGCTTCA	
KA002375-Common	TCCTTCTGACCAAGCATATGATCA	
KA002376-X	GAAGGTGACCAAGTTCATGCTTCAGAGACATGATCCTCAACCTTC	Chr4
KA002376-Y	GAAGGTCGGAGTCAACGGATTTCAGAGACATGATCCTCAACCTTG	
KA002376-Common	GTCGCTCACTTCTTTGATGTTTCT	
KA002381-X	GAAGGTGACCAAGTTCATGCTAGTTTCACTGTCTACATCATCCCA	Chr10
KA002381-Y	GAAGGTCGGAGTCAACGGATTAGTTTCACTGTCTACATCATCCCT	
KA002381-Common	GCAGAGGCATTTCTATAAGTGAGC	
KA002388-X	GAAGGTGACCAAGTTCATGCTAACCGTTGGCTCTTATATGCATAC	Chr10
KA002388-Y	GAAGGTCGGAGTCAACGGATTAACCGTTGGCTCTTATATGCATAT	
KA002388-Common	CTTCCGAATGCTGCTTAGTTATCG	

KA002391-X	GAAGGTGACCAAGTTCATGCTGTGTATGTTGGGCAGTTTAACCG	Chr10
KA002391-Y	GAAGGTCGGAGTCAACGGATTGTGTATGTTGGGCAGTTTAACCA	
KA002391-Common	TGACTACCCACATGCATACAGAAA	
KA002397-X	GAAGGTGACCAAGTTCATGCTTGGTATTAGCTAATATCTCCCATTTGG	Chr10
KA002397-Y	GAAGGTCGGAGTCAACGGATTTGGTATTAGCTAATATCTCCCATTTGC	
KA002397-Common	TGTCTTTGTGTATTTAATCCGTGGT	

Table S4. Primers for Sequence Analysis

Gene ID	Annotation	Sequence
EGP22363	Promoter	F: ACTAAGTATGGGAAAGTATGCTATC
		R: GGACTGAGTACCCCTTGACCT
	DNA	F: GCATCCAGGTCTGACTTTAATAATCT
		R: TGGGACAATAGGCACTCTGAAC

Table S5. Primers for Real-time PCR.

Gene ID	Gene name	Sequence
EGP22363	*SmANS*	F: ACTAAGTATGGGAAAGTATGCTATC
		R: GGACTGAGTACCCCTTGACCT
EGP02608	*SmAPRT*	F: TGGCGCCTCATGATCCGATTCTTA
		R: ACTCCAACACGCTCAAGAAGCCTA
EGP21875	*SmMYB75*	F: AACAAGTGACAAGCAAACTACCGATG
		R: CACATTGTTCTTGTTGCATGGAGTT
EGP18807	*SmJAF13*	F: TCAATATGATCGATAAGGAAGTCTCG
		R: ATTGTTCCAACTGCTACTGTTTTCG
EGP10955	*SmWD40*	F: ATGCCTGTGGCTGAGTTGGAAAG
		R: CGTTGTGCAGCAGACCACTGAATT

Table S6. Phenotype of fruit peel color in 42 eggplant inbred lines.

No.	Phenotype	No.	Phenotype

1	green	22	purple
2	green	23	green
3	green	24	green
4	green	25	purple
5	green	26	purple
6	green	27	purple
7	purple	28	green
8	purple	29	purple
9	purple	30	purple
10	purple	31	purple
11	purple	32	purple
12	purple	33	purple
13	purple	34	purple
14	purple	35	purple
15	purple	36	purple
16	purple	37	purple
17	green	38	purple
18	purple	39	purple
19	purple	40	purple
20	purple	41	purple
21	green	42	purple

Table S7. Phenotype of fruit peel color in 24 eggplant hybrid varieties.

No.	Phenotype	No.	Phenotype
1	green	13	purple
2	green	14	purple
3	purple	15	purple
4	purple	16	purple
5	purple	17	purple

**6**	**green**	**18**	**purple**
**7**	**green**	**19**	**purple**
**8**	**green**	**20**	**purple**
**9**	**purple**	**21**	**green**
**10**	**green**	**22**	**green**
**11**	**purple**	**23**	**purple**
**12**	**purple**	**24**	**purple**

Figure S1. Relative expression levels transcription factors related to anthocyanin synthesis. Asterisks indicate significant differences, according to Student's t-test (P < 0.01). The error bar indicates the standard deviation

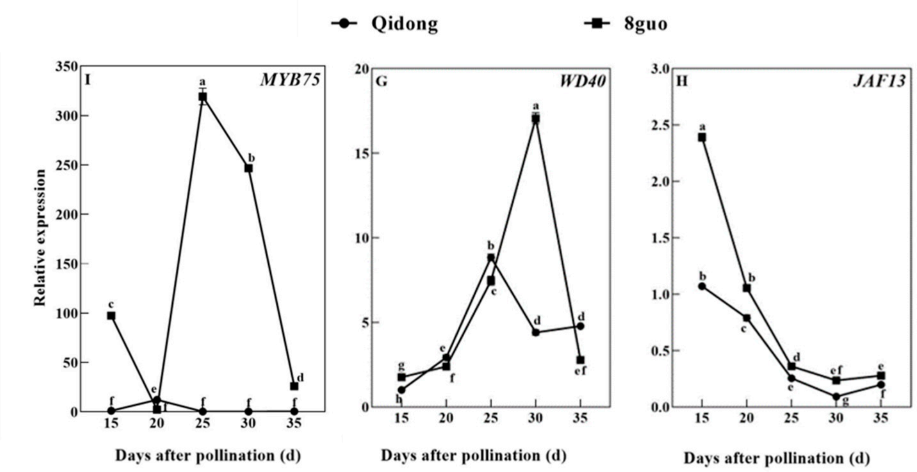

Figure S2. Sequence of *EGP22363* from 'Qidong' and '8 guo' . The SNP are in italic and marked by red .

CDS Sequence of '8 guo'

ATGGCTGAAGTAGTTTCCAAATCAGTACAAGAACTTGCAAACAACAACCA

AGTTCCAGAAAATTATATTCATTCGCAAGGGTCAATCAGTAGCTCTTCTC

CCCTGCTTGATGTGCCACAAGTTGATCTTAGCCTTCTCATATCACCAACA

TCCCCTGCCAGACAACAAGAGCTCAACAAACTTCAATCAAACCTCAAGTT

TTGTGGCTGCATCCAGGTCATAAATCATGGAATGACAGATTCATTTCTTG

ACAAAGTGCGCGAAATTAGCAAGCAATTCTTTGCTCTTCCAACTGAAGAG
AAGCTTAAATATGGCAGAACAGTTGATGGCATTGAAGGATACGGAAGTGA
TAAAGTTCTTAACGATAAGCAAAGGCTTGATTGGTCTGATAGATTCTACC
TAAATGTGTTCCCTGAAGATATAAGAATACTCCAATTATGGCCCCAAAAA
CCTAAATGTTTTAGAAAAGTTTTGGAAGAATATATCAAGAATATGAAGTT
ATTGAATGAGTCCCTCCTAAAGGCCATGGCATCGT*T*ATTGAACCTGAAGG
AGAATTGCTTTCTGGACTAG

CDS Sequence of ' Qidong '

ATGGCTGAAGTAGTTTCCAAATCAGTACAAGAACTTGCAAACAACAACCA
AGTTCCAGAAAATTATATTCATTCGCAAGGGTCAATCAGTAGCTCTTCTC
CCCTGCTTGATGTGCCACAAGTTGATCTTAGCCTTCTCATATCACCAACA
TCCCCTGCCAGACAACAAGAGCTCAACAAACTTCAATCAAACCTCAAGTT
TTGTGGCTGCATCCAGGTCATAAATCATGGAATGACAGATTCATTTCTTG
ACAAAGTGCGCGAAATTAGCAAGCAATTCTTTGCTCTTCCAACTGAAGAG
AAGCTTAAATATGGCAGAACAGTTGATGGCATTGAAGGATACGGAAGTGA
TAAAGTTCTTAACGATAAGCAAAGGCTTGATTGGTCTGATAGATTCTACC
TAAATGTGTTCCCTGAAGATATAAGAATACTCCAATTATGGCCCCAAAAA
CCTAAATGTTTTAGAAAAGTTTTGGAAGAATATATCAAGAATATGAAGTT
ATTGAATGAGTCCCTCCTAAAGGCCATGGCATCGT*C*ATTGAACCTGAAGG
AGAATTGCTTTCTGGACTAG
